# Open Trans-Scaphoid Transcapitate Perilunate Fracture-Dislocation: A Case Report

**DOI:** 10.7759/cureus.38958

**Published:** 2023-05-13

**Authors:** Abdulrahman J Korkoman, Sara Alrajhi, Abdulaziz A AlQahtani

**Affiliations:** 1 Orthopedic Surgery, University of Bisha, Bisha, SAU; 2 Orthopedic Surgery, Prince Sultan Military Medical City, Riyadh, SAU; 3 Orthopedics, King Fahad Specialist Hospital, Riyadh, SAU

**Keywords:** management scaphoid fracture, wrist surgery, perilunate injuries, scaphoid fractures, wrist injuries, wrist dislocation, mayo wrist score, lunate dislocation, wrist fractures

## Abstract

Perilunate dislocations and fracture-dislocations are considered rare injuries. Perilunate injuries are frequently missed during primary evaluations. We report a case of a 37-year-old male presenting with an open perilunate fracture-dislocation a few days after experiencing trauma. He underwent repeated debridements, and provisional external fixator application followed by a definitive open reduction through a combined dual approach and internal fixation of scaphoid and capitate with headless screws. Aggressive physiotherapy exercises were started eight weeks after definitive fixation. After six years, the patient had a satisfactory outcome with an excellent Mayo wrist score. Perilunate injuries should be considered one of the important differential diagnoses in wrist injuries. Early diagnosis and treatment are of utmost importance to gain optimum outcomes. The best results could be achieved with open reduction and internal fixation through a combined volar and dorsal approach.

## Introduction

The wrist joint is made up of the distal ends of the radius and ulna, eight carpal bones, and five proximal bases of the metacarpals. The carpal bones are further divided into proximal and distal rows; the proximal row contains the scaphoid, lunate, triquetrum, and pisiform while the distal row contains the trapezium, trapezoid, capitate, and hamate [1]. The articulation of the carpal bones and the ligamentous attachments enables wrist motion in flexion-extension and radio-ulnar deviation [2].

Perilunate dislocation and perilunate fracture-dislocations are relatively rare injuries that account for around 7% of all carpus injuries [3]. It is often overlooked in primary evaluations, A retrospective multicentre study showed that around 25% of perilunate injuries were missed [4]. As perilunate injuries are commonly missed, a high index of suspicion is warranted, and early and correct diagnosis of such injuries is of paramount importance to regain functional wrist range of motion and limit chronic wrist pain and discomfort [5]. Perilunate open injuries account for less than 10% of all perilunate injuries [4]. They usually result from high-energy mechanisms that are usually associated with trauma to other organ systems in 26% of cases and to ipsilateral limbs in 11% of cases [4,5]. 

The mechanism of perilunate dislocations was first introduced by Mayfield et al. in their 1980 study, and it usually involves an axial load to a hyperextended ulnar deviated wrist with intercarpal supination that would lead to the sequential failure pattern around the lunate, which is termed “progressive perilunar instability” [6]. The dislocation could either course through a greater arc, which involves ligamentous disruption associated with fractures, or a lesser arc, which pertains to a pure ligamentous injury. There are multiple variants of the greater-arc injuries, which could be classified into either an intact scaphoid (transradial-styloid, transcapitate, transtriquetrum, and combinations) or a trans-scaphoid (trans-scaphoid transradial-styloid, trans-scaphoid transcapitate, trans-scaphoid transtriquetrum, and combinations) [4,6].

Mayfield et al. [6] demonstrated in their study that the slower the application of force the more the fracture variants termed “greater arc injuries” described above are likely to happen. While more rapid application of axial load means pure ligamentous injuries, “lesser arc injuries” are more likely to happen. This article reports a case of an adult male with a rare type of perilunate injury. We describe the presentation, radiographic findings, surgical approaches, reduction and stabilization principles, and clinical and radiographic outcomes.

## Case presentation

**Figure 1:**
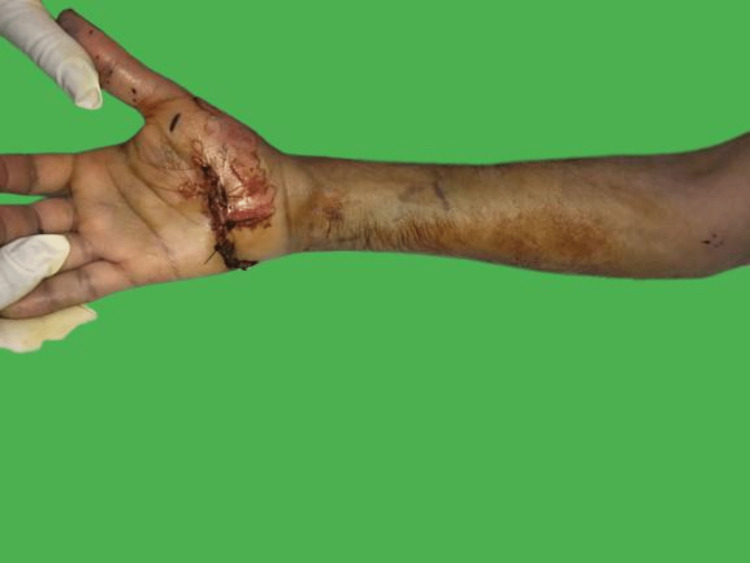
5-cm palmer wrist wound over the carpus

A fit and well 37-year-old male, a right-handed non-smoker who works as a soldier was driving his motorbike at high speed when he lost control and fell directly on his outstretched right hand on a highway. He went to a nearby hospital where initial resuscitation was offered; a palmar wrist wound of around 5 cm over the carpus was locally irrigated and sutured in the trauma bay and he was advised to go to a higher center (Figure 1).

**Figure 2:**
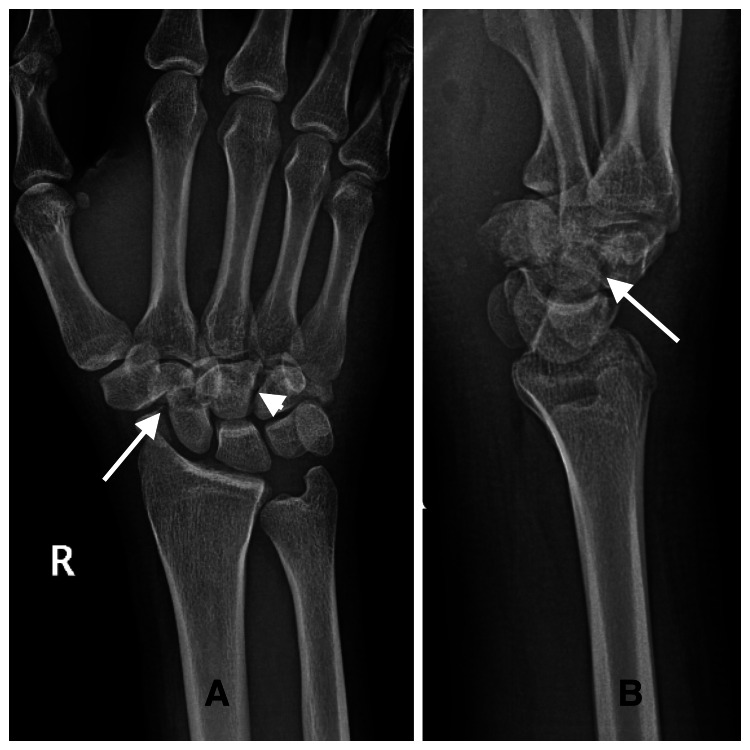
Right wrist X-rays showing break of Gilula’s lines, loss of colinearity of radius, lunate, and capitate, squared capitate, and distal pole scaphoid fracture (A) Anteroposterior and (B) lateral views of the right wrist (arrows show the scaphoid fracture, and the arrowhead shows the capitate fracture)

The patient presented to our outpatient fracture clinic where his clinical examination showed a 5-cm sutured wound over the right palmar wrist with no features of wound infection, generalized palmar, and dorsal wrist tenderness while the range of motion was limited by wrist pain. The findings of the neurovascular examination were normal. Plain wrist radiography showed a break of Gilula’s lines, loss of colinearity of radius, lunate, and capitate, squared capitate, and distal pole scaphoid fracture (Figure 2).

**Figure 3:**
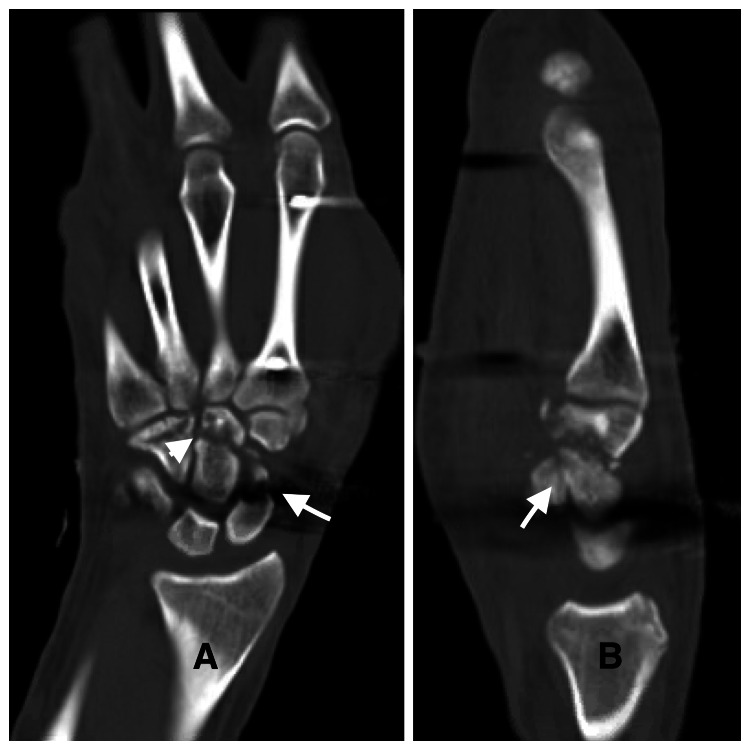
CT scan of right wrist showing right carpal bones fractures after external fixator application (A) Coronal and (B) sagittal reconstructed CT images (arrows show the scaphoid fracture, and the arrowhead shows the capitate fracture) CT: computed tomography

The patient was admitted, started on intravenous antibiotics, and underwent irrigation and debridement, and an external fixator was applied. For surgical planning, a wrist CT was performed (Figure 3).

Two days later, the patient underwent another round of irrigation and debridement. Then, definitive open reduction and internal fixation of the perilunate injury was done through combined standard volar and standard dorsal approaches with two headless cannulated screws in the scaphoid, three headless cannulated screws in the capitate, and carpal tunnel decompression. The patient was fitted with a volar slab in 20 degrees of wrist extension for eight weeks and the plaster slab was discontinued and replaced by a removable wrist splint until 12 weeks. Postoperative plain radiographs showed normal carpal bone alignment with a scapholunate angle of 48 degrees and a gap of 2 mm.

**Figure 4:**
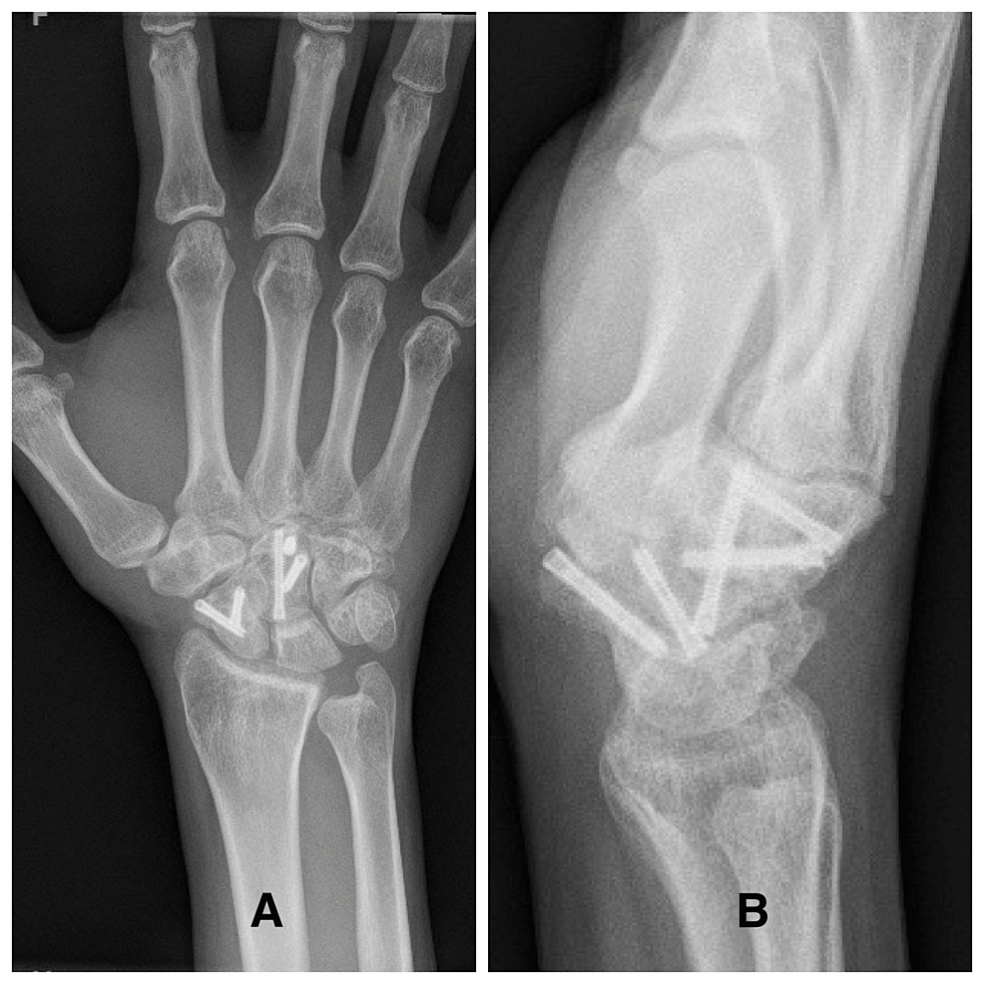
Right wrist X-rays showing open reduction of the fractures and internal fixation at six years postoperatively (A) Anteroposterior view. (B) Lateral view

Thereafter, passive and active range of motion started sequentially, followed by grip-strengthening exercises. The patient was followed up for six years (Figure 4).

**Figure 5:**
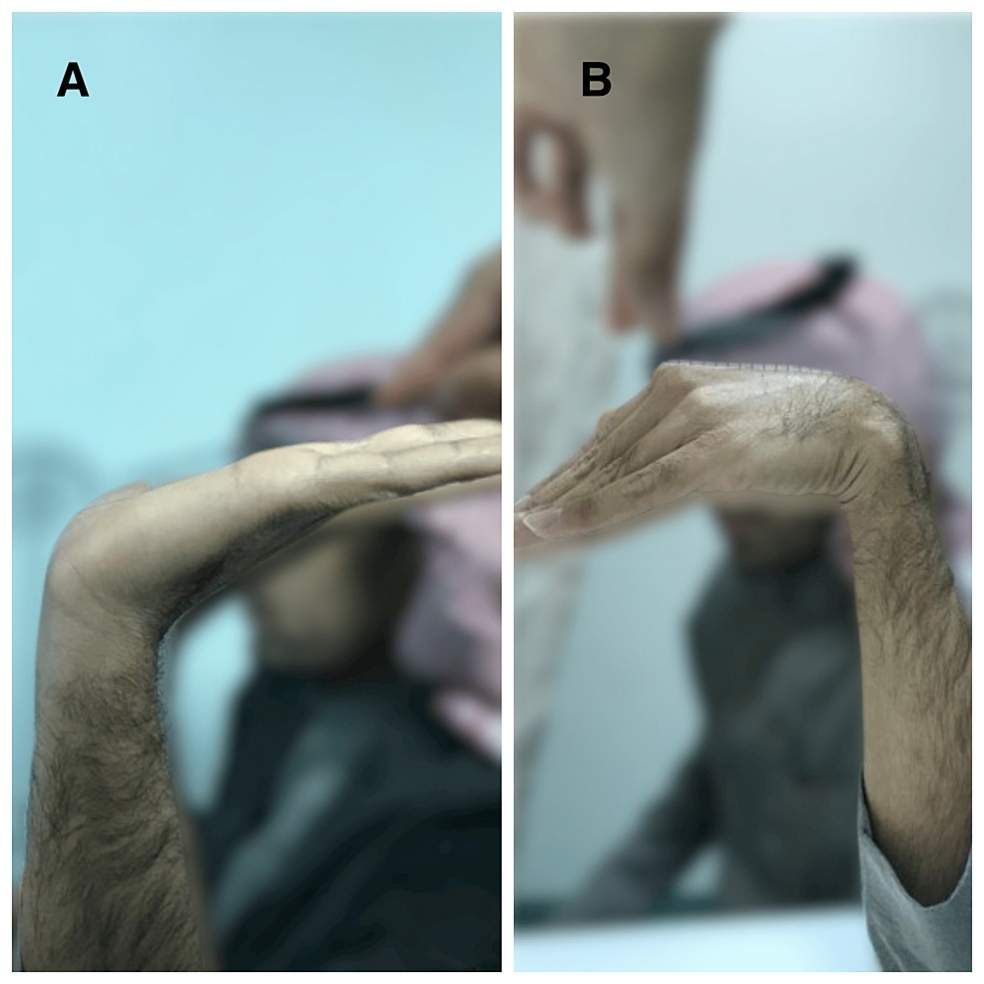
Right wrist range of motion at six years postoperatively (A) Extension. (B) Flexion

The patient had mild, occasional wrist pain. He returned to his regular work with 90% of grip strength (compared to the opposite non-injured side), an extension of 80°, flexion of 75°, radial deviation of 20°, and ulnar deviation of 40°. The Mayo wrist score was 90 (excellent) (Figure 5) [7].

## Discussion

Carpal bones and ligaments act as links between the radius, distal ulna, and metacarpals and are essential for the stability of the wrist. Patients with perilunate dislocations or fracture-dislocation usually present with wrist pain, swelling, and paresthesia over the median nerve distribution. Hence, a thorough neurovascular examination of the extremity is important [3]. Proper biplane imaging is essential to avoid a missed diagnosis; traction views might help in a better understanding of the fracture pattern, and CT is helpful for surgical planning and should be done after close reduction [8].

Open perilunate dislocation or fracture-dislocation is an orthopedic emergency, and hence debridement, carpal tunnel release, and external fixator application, if there is unstable carpus, should be done emergently. A better understanding of the mechanism of injury and the subsequent extent of the injury has led to better management strategies. Closed manipulative reduction and immobilization do not usually restore and maintain carpal alignment and have poor results [3,9]. Operative intervention has become the gold standard of management with a combined approach showing good to excellent results [10]. However, there are multiple areas of debate and conflict, which include the timing to open surgical intervention in the absence of acute carpal tunnel syndrome, the approach to be utilized, how and which ligament to fix, and the best fixation construct. There are three approaches for perilunate injuries: volar, dorsal, and a combined dual approach (which was used in this case). Each has its own advantages and disadvantages. The volar approach is done through an extended carpal tunnel incision in line with the ulnar border of the palmaris longus tendon. It allows for carpal tunnel release, reduction of lunate, repair of the volar lunotriquetral ligament and scapholunate ligament, and repair of the capsule at the space of Poirier [8]. The dorsal approach is done through a longitudinal incision over Lister’s tubercle down to the extensor retinaculum with ulnar and radial skin flaps, and then the retinaculum is opened in line with the third dorsal compartment. It usually helps in fixing carpal fractures, giving the exposure needed for alignment restoration and repair of the scapholunate interosseous ligament, which affects long-term functional scores [8,11].

Combined dual dorsal and volar approaches provide the advantages of both above-mentioned approaches, and hence it is usually preferred by many surgeons [10]. Fixation of fractured carpal bones can be done either with Kirschner wires or headless cannulated compression screws with the latter being more advantageous as they provide compression across the fracture site, allow an earlier return of range of motion, and withstand a higher load [12].

The cut-off for delayed diagnosis is four weeks, after which the perilunate injury is considered missed. For young patients aged less than 30 years, a reduction and reconstruction are still feasible and advisable, while in older patients, it usually requires a salvage procedure like proximal row carpectomy due to the poor outcome and functional scores associated with reconstruction procedures [8]. Even in cases of a perfect restoration of carpal alignment, complete bone healing, and full ligamentous healing, a normal wrist should not be expected. The goals of management are stable carpus, as minimal pain as possible, and a functional wrist range of motion [8].

In our case, a stable wrist and complete fracture healing were achieved, and no signs of avascular necrosis or post-traumatic carpal arthritis were observed.

## Conclusions

Perilunate injuries should be considered one of the important differential diagnoses in wrist injuries. Early diagnosis of perilunate injuries is critical for achieving the best outcomes. The treatment should aim to restore carpal alignment and either repair or reconstruct intercarpal or radiocarpal ligaments. A combined dual approach gives better exposure to achieve the goals of treatment. However, even with perfect reduction, regaining a fully normal wrist is less likely but a functional wrist can be achieved.
